# Emerging trends in cervical cancer incidence among younger Taiwanese generations: an urban–rural comparison

**DOI:** 10.1080/07853890.2025.2458765

**Published:** 2025-01-30

**Authors:** Ya-Xuan Wu, Yi-Chu Chen, Jing-Rong Jhuang, Chun-Ju Chiang, Wen-Chung Lee

**Affiliations:** a College of Public Health, Institute of Health Data Analytics and Statistics, National Taiwan University, Taipei, Taiwan; b College of Public Health, Institute of Epidemiology and Preventive Medicine, National Taiwan University, Taipei, Taiwan; c Taiwan Cancer Registry, Taipei, Taiwan

**Keywords:** Cervical cancer, cohort effect, secular trend, urbanization, sexual activity

## Abstract

**Background:**

Despite global declines in cervical cancer incidence, certain regions observe unexpected rising trends among younger generations.

**Methods:**

This study uses the age–period–cohort model to examine long-term incidence trends of invasive cervical cancer in Taiwan. Data were sourced from the Taiwan Cancer Registry.

**Results:**

From 2000 to 2019, both urban and rural areas of Taiwan saw a marked decrease in incidence rates, a trend largely attributed to the cytology-based screening program introduced in 1995. Yet, rising incidence rates emerged in younger Taiwanese generations, specifically those born post-1975 in urban and post-1980 in rural settings. The 1990-born urban cohort faced a risk 1.9 times higher than their 1975-born counterparts, while the risk for the 1990-born rural cohort was 1.4 times greater than those born in 1980. In addition, post-1980 urban cohorts exhibited greater risks than their rural counterparts.

**Conclusions:**

The rising trends in cervical cancer incidence among younger Taiwanese generations may be influenced by factors such as increased sexual permissiveness and urbanization. Although current prevention efforts, such as human papillomavirus vaccination, are noteworthy, there is a need for ongoing surveillance and improved strategies that specifically target recent cohorts.

## Highlights

Cytology screening in Taiwan has lowered cervical cancer rates.

Younger cohorts in Taiwan show rising cervical cancer risks.

Urban–rural disparities exist in cervical cancer trends across birth cohorts.

Urbanization and changes in sexual behaviour influence cervical cancer trends.

HPV vaccination since 2018 covers those born after 2004, leaving a gap for earlier births.

## Introduction

Cervical cancer ranks as the fourth most diagnosed cancer and is the fourth leading cause of cancer-related deaths among women globally [1]. Although the past few decades have witnessed a significant decline in both mortality and incidence rates of cervical cancer in several countries, this trend is predominantly observed in high-income regions such as Europe and the United States. Conversely, in countries with a lower Human Development Index, cervical cancer persists as the most frequently diagnosed cancer [2,3]. Recognizing the gravity of this public health concern, the World Health Organization (WHO) in 2020 issued a call for the worldwide elimination of cervical cancer, setting an objective to curtail the global annual age-stand­ardized incidence to four cases per 100,000 woman-years [4].

Human papillomavirus (HPV) infection, a sexually transmitted disease, is considered a necessary but not sufficient condition for the development of cervical cancer [5]. The advent of effective HPV vaccines [6] and screening programs has rendered cervical cancer largely preventable. As a result of organized screening initiatives, many countries have observed a decreasing trend in cervical cancer incidence rates [7–9]. Yet, some high-income countries have noted a rise in incidence among younger generations [7,8,10]. Taiwan initiated a nationwide cervical cancer screening program in 1995, reducing the age-standardized incidence rate from 25.2 per 100,000 woman-years in that year to 7.8 per 100,000 woman-years by 2019. Despite this decrease, as of 2019, cervical cancer remains the ninth most frequently diagnosed cancer and the eighth leading cause of cancer-related mortality among women in Taiwan [11].

In secular trend analysis, age-standardized incidence rates can become confounded by influences attributed to the period (calendar year) and the birth cohort (generation). In this study, we employ the age–period–cohort model [12,13] to assess the long-term trends of cervical cancer incidence in Taiwan. Through this approach, we elucidate the obscured reversal in the birth-cohort trend and identify disparities between urban and rural areas in the birth-cohort effect.

## Methods

In this study, cases of invasive cervical cancer (C50) were identified using the third revision of the International Classification of Diseases for Oncology (ICD-O-3). Diagnostic methods for cervical cancer cases were almost exclusively based on cytology and histopathology (Appendix 1). These case data were sourced from the Taiwan Cancer Registry, a nationwide population-based registry renowned for its high-quality data [14,15]. Data from patients under 25 years of age at diagnosis were excluded due to the scarcity of such cases (Appendix 2). Population data for Taiwanese women were obtained from the Department of Statistics under the Taiwan Ministry of the Interior. Age-standardized incidence rates for women aged 25 and above were calculated using the WHO 2000 World Standard Population. All townships in Taiwan were categorized into one of seven urbanization levels based on criteria such as population density, educational attainment, the proportion of the elderly population, the ratio of the agricultural population, and the availability of medical resources [16]. The first two levels of urbanization were designated as urban areas, with the remaining ones classified as rural. The study incorporated data from 69 urban and 280 rural townships, with townships from Taiwan’s outlying islands being excluded.

The age–period–cohort model was used to analyse and decompose overall trends into three components: age effects, reflecting risk variations across the human lifespan; period effects, representing the impact on all age groups within a specific period; and cohort effects, capturing risk differences between birth cohorts. The age–period–cohort model is inherently nonidentifiable due to the perfect collinearity among its three temporal variables (cohort + age = period) [12]. We utilized the method presented by Rosenberg et al. [13] which focuses on the estimable parameters without disentangling the three effects: (1) longitudinal age relative risks (adjusted for cohort and period deviations), (2) period relative risks (adjusted for age and cohort deviations) and (3) cohort relative risks (adjusted for age and period deviations). Throughout this paper, these parameters are referred to as the age, period and cohort effects, respectively.

The dataset was stratified into 13 age groups (25–29, 30–34, … and over 85; cases below age 25 were excluded due to small numbers). This five-year grouping was chosen to ensure a sufficient number of cases in each category. In the age–period–cohort analysis, given that age was grouped in five-year intervals, periods were similarly grouped into four five-year intervals (2000–2004, 2005–2009, … and 2015–2019). This categorization led to the definition of 16 birth cohorts (mid-year: 1915, 1920, …, 1990). Data management and analyses were performed using SAS (version 9.4; SAS Institute, Cary, NC). The age–period–cohort model was analysed using the APC web tool (https://analysistools.nci.nih.gov/apc/) [13]. We considered a two-sided *p* value of <.05 as statistically significant.

## Results

**Figure 1.**
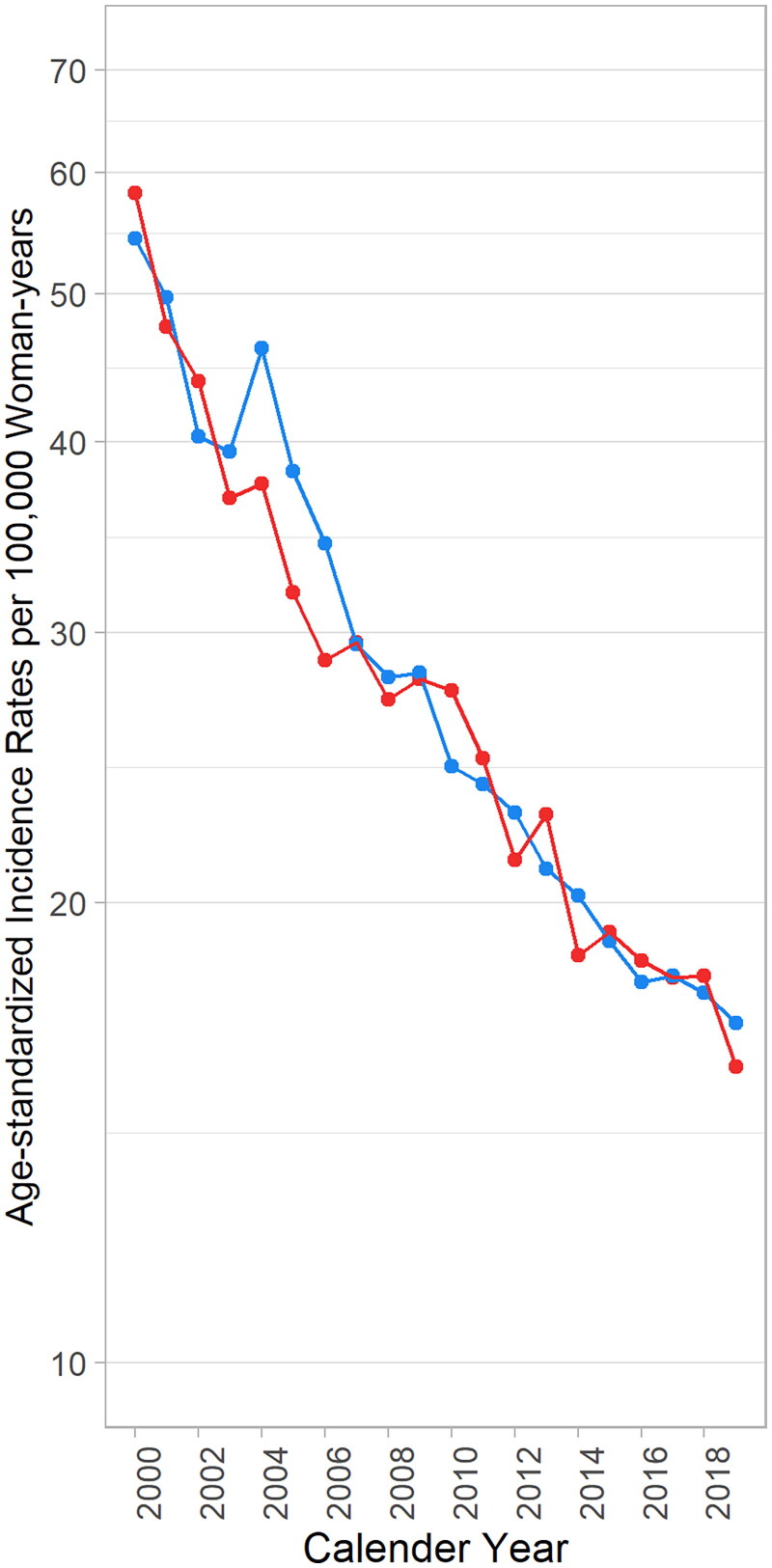
Age-standardized incidence rate of cervical cancer from 2000 to 2019 in women in Taiwan (red: urban region; blue: rural region).

Figure 1 depicts the secular trends of age-standardized incidence rates for invasive cervical cancer among Taiwanese women. Both urban and rural areas witnessed a decline: In urban areas, the rate decreased from 58.2 per 100,000 woman-years in 2000 to 15.6 in 2019 (a 3.7-fold reduction), while in rural areas, it fell from 54.3 to 16.7 per 100,000 woman-years (a 3.3-fold reduction). Remarkably, the trends in both areas paralleled each other throughout the studied periods.

**Figure 2.**
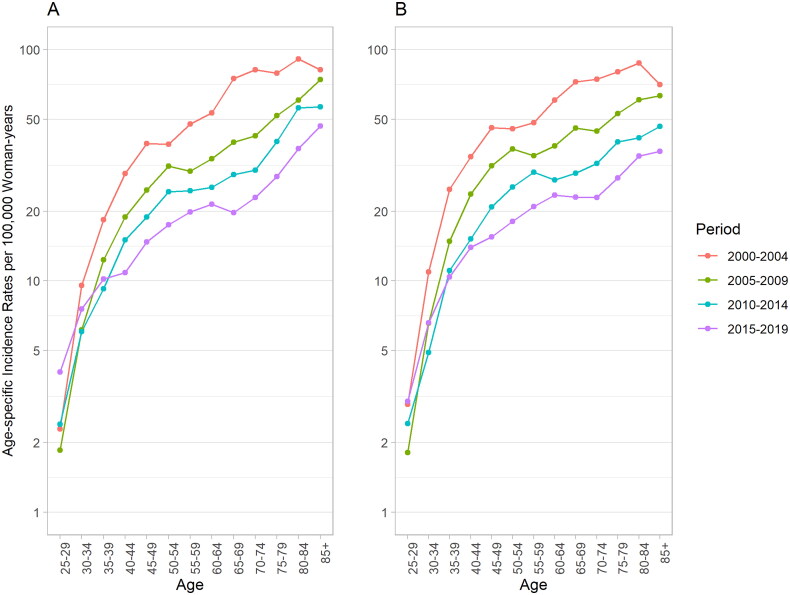
Cross-sectional age curves of cervical cancer incidence rates in women in Taiwan (A: urban region; B: rural region).

Figure 2 presents the cross-sectional age curves of cervical cancer incidence in women in Taiwan. We observed an increasing risk of cervical cancer with advancing age in urban and rural areas across the studied periods. From 2000 to 2019, in urban areas, the rate shows a sharp rise before the 45–49 age group, increasing from an average of 2.6 per 100,000 woman-years in the 25–29 age group to 24.4 in the 45–49 age group (a 9.4-fold increase), followed by a more gradual increase to 64.8 in the 85 and above age group (a 2.7-fold increase). Similarly, the rate in rural areas shows a comparable pattern: rising from 2.5 to 28.5 per 100,000 woman-years (an 11.4-fold increase), then gradually increasing to 54.2 (a 1.9-fold increase). Furthermore, we observed the curves after the 40–44 age group decline with advancing time periods in both urban and rural areas.

**Figure 3.**
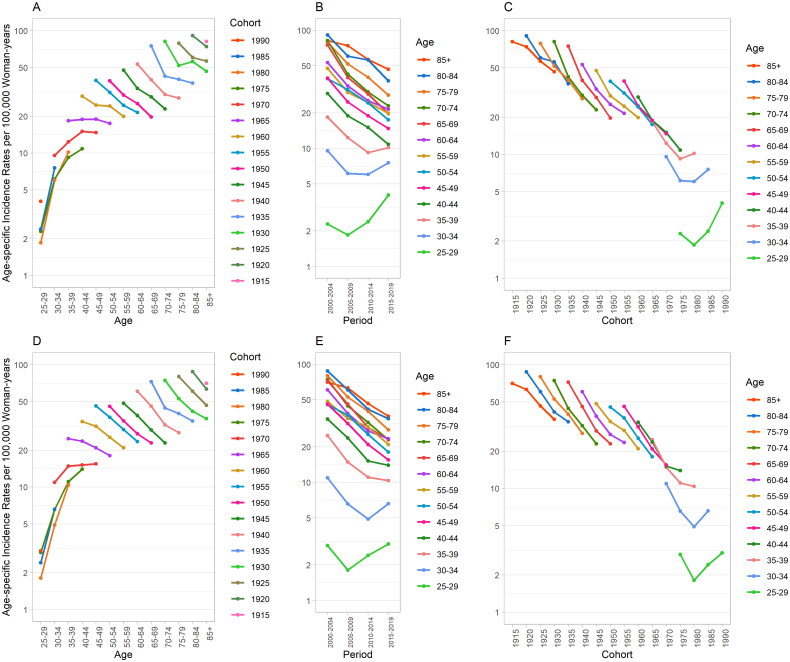
Age-specific incidence rate of cervical cancer in women in Taiwan by age, period and cohort (urban region: A, B, C; rural region: D, E, F).

Figure 3 presents the age-specific incidence rates of cervical cancer in women in Taiwan by age, period and cohort. A notable pattern emerges in the same birth cohort: incidence rates rise sharply before age 40–44 and then decline (Figure 3(A,D)). The period trends, on the other hand, demonstrate variation across different age groups (Figure 3(B,E)). For women aged 40–44 and above, incidence rates in both urban and rural areas decrease over time. However, for the 30–34 age group, the urban area’s declining incidence rates slowed between 2005–2009 and 2010–2014 before increasing; the rural area’s rates fell more sharply before rising. In the 25–29 age group, the decline in incidence rates ceased during 2005–2009 in both urban and rural areas and was followed by an increase. Regarding birth-cohort trends, a declining trend in earlier cohorts for both urban and rural areas was noticeable. However, these trends reversed, beginning with the post-1975 cohort in urban areas and the post-1980 cohort in rural areas, where the incidence rates started to rise (Figure 3(C,F)).

**Figure 4.**
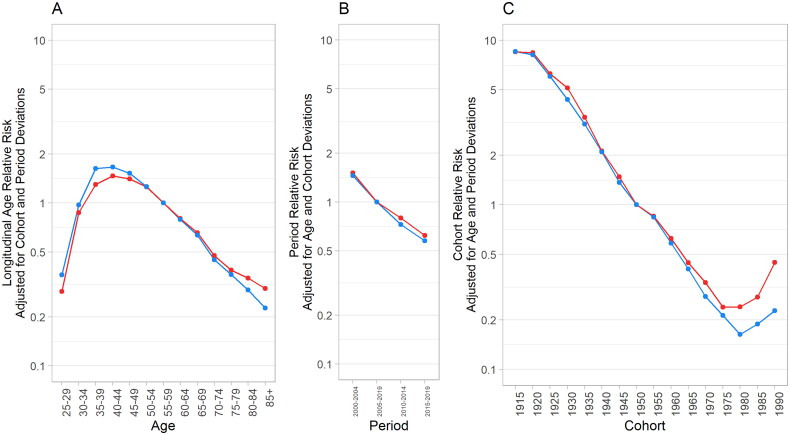
Effects of age, period and cohort on cervical cancer incidence rates in women in Taiwan (red: urban region; blue: rural region).

Figure 4 illustrates the results of the age–period–cohort analysis for cervical cancer incidence among Taiwanese women. The age effects reveal a notable increase in cervical cancer risk with advancing age in urban and rural areas, peaking at ages 40–44 and then declining (Figure 4(A)). The period effects demonstrate a consistent downward trend in urban and rural areas from 2000 to 2019 (Figure 4(B)). Regarding the cohort effects (Figure 4(C)), urban and rural areas exhibited a declining trend in cohorts born before 1970. However, a distinct reversal of this declining trend occurred in the post-1975 cohort in urban areas, displaying an increasing risk. The risk for cohorts born in 1990 was 1.9 times higher than those born in 1975. Similarly, rural areas experienced a trend reversal, albeit beginning five years later (post-1980 cohort). The risk for cohorts born in 1990 was 1.4 times higher than those born in 1980. Notably, significant disparities in cervical cancer risks between urban and rural areas emerged in cohorts born after 1980, with the risk for cohorts born in 1980 being 1.5 times higher in urban areas than in rural areas.

## Discussion

The study conducted on cervical cancer in Taiwan revealed several key findings. First, there was a significant decline in age-standardized incidence rates of cervical cancer in both urban and rural areas between 2000 and 2019. Cross-sectional age analysis showed an increased risk of cervical cancer with age in all areas. A unique pattern was observed in the same birth cohorts, with incidence rates peaking before age 40–44 and then declining. Period trends varied across age groups, with a general decrease over time, but an increase in incidence rates was noted in younger age groups in recent periods. The study also observed a reversal in the declining trend of cervical cancer risk among younger generations, especially in urban areas, likely due to changes in sexual behaviour and heightened susceptibility to HPV infection. Despite the overall decline in incidence rates, the research highlighted a persistent and increasing risk in more recent cohorts, especially in urban settings compared to rural areas.

This study identifies an upward trend in cervical cancer risk among younger generations in Taiwan, a pattern that mirrors observations in other nations such as European countries and Japan [8]. This cohort effect might be linked to heightened sexual permissiveness in recent generations, often marked by earlier sexual initiation and having multiple sexual partners. These factors increase vulnerability to HPV infection, subsequently elevating the risk of cervical cancer [8,17–19]. Supporting this, a 2000 survey by the Taiwan Department of Health highlighted a rise in sexual permissiveness among the nation’s youth [20]. Moreover, we observed disparities in the cohort effect trends between urban and rural areas. Urban regions saw a reversal in the declining trend about five years before their rural counterparts. Research indicates that areas undergoing more rapid modernization often report more liberal sexual behaviours among their young populace [21]. Given Taiwan’s swift urbanization and modernization over the past 50 years, coupled with cultural evolution, these factors could be driving the observed discrepancies in the cervical cancer cohort effect between urban and rural settings. Intriguingly, urbanization in Taiwan influences the cohort effect for another malignancy – female breast cancer – though in a contrasting manner [22].

This study highlights a notable decrease in the age-standardized incidence rate and a declining period effect of cervical cancer in Taiwan. This reduction is largely attributed to the organized cytology-based screening program introduced in Taiwan in 1995 [23]. Similar declines in cervical cancer incidence due to the implementation of screening programs have been observed in many countries [8,17], with some low- and middle-income countries without effective screening initiatives being the exception [8,24,25]. As mentioned previously, WHO has set a goal for the global elimination of cervical cancer [4]. It is worth noting that cytology-based screening tends to be more effective against cervical squamous cell carcinoma than adenocarcinoma. In many countries, there have been a more pronounced declining period effect for squamous cell carcinoma compared to adenocarcinoma [26,27]. Our study reports similar findings (Appendices 3 and 4). Conversely, when examining the cohort effect, there was a more pronounced increase in adenocarcinoma compared to squamous cell carcinoma in recent cohorts (Appendix 4). Despite 82% participation among Taiwanese women since 1995, only 54% of women aged 30–69 adhere to triennial screenings, with even lower rates among older women. Strategies such as education, reminders and incentives aim to boost uptake [23,28,29]; yet, compliance was only 43.5% in 2016, underscoring the need for improved access [30]. HPV-based screening, which offers 70% greater protection against invasive cancer compared to cytology [29], is now being considered in Taiwan after 30 years of cytology use.

Despite WHO recommendations, only a few high-income countries have achieved HPV vaccination coverage above 90% for the target age group [31]. Barriers include vaccine supply shortages [31], budgetary constraints [32] and concerns about vaccine side effects [33]. Taiwan’s HPV vaccination program, launched in 2018, has been notably successful, with coverage rates of 76.8% in 2018 and 86.9% in 2019, supported by its robust healthcare infrastructure [34]. Previous research using macrosimulation methods estimated that if Taiwan’s HPV vaccination coverage reaches 90%, the country could achieve cervical cancer elimination by 2050 [35]. However, this strategy primarily protects individuals born after 2004 from HPV-related diseases [6]. Our study’s data only include individuals born up to the 1990 cohort. If the current trend continues, women born between 1990 and 2004 may have an increased risk of cervical cancer. For the 1990–2004 birth cohorts, enhanced cervical cancer prevention education is crucial. The primary control measure is cervical screening, complemented by education on risk factors such as early sexual debut, multiple sexual partners, non-barrier contraception and smoking.

In Taiwan, the cross-sectional age curve of cervical cancer generally shows a rise with increasing age, lacking a clear age peak. However, when employing the age–period–cohort analysis and adjusting for period and cohort deviations, we identified a peak age effect at ages 40–44, which then consistently declines. The natural history of cervical cancer progression shows distinct age-related peaks: HPV acquisition during adolescence and early adulthood, and cervical cancer between ages 45 and 60. This trend is attributed to increased sexual activity in youth, leading to HPV infections that, after a 30-year latency, peak as cervical cancer at ages 40–44 [36].

The study on cervical cancer incidence in Taiwan demonstrated notable strengths and weaknesses. Among its strengths was the comprehensive use of the age–period–cohort model, which provided a nuanced understanding of trends and allowed for the distinction between age, period and cohort effects. The study’s extensive dataset, sourced from the Taiwan Cancer Registry, offered a robust foundation for analysis. Additionally, the stratification of data into detailed age, period and urbanization categories enabled a precise examination of trends across different demographics. However, the study also had limitations. The age–period–cohort model, while insightful, is constrained by the issue of nonidentifiability, meaning certain effects cannot be uniquely determined due to inherent collinearity among age, period and cohort. Another limitation was the study’s focus on invasive cervical cancer, potentially overlooking trends in pre-invasive stages of the disease. Our data only includes International Federation of Gynecology and Obstetrics (FIGO) staging from 2004 onwards (Appendix 5), making it impossible to conduct an age–period–cohort analysis by FIGO stage for the entire study period (2000–2019). Additionally, the database lacked important variables influencing cervical cancer, such as HPV infection type, number of sexual partners, pregnancy and childbirth, precluding a more in-depth analysis. The exclusion of patients under 25 might have also impacted the understanding of the disease’s onset. Lastly, the study was limited to Taiwan, and its findings might not be generalizable to other populations with different socio-cultural and healthcare contexts.

The findings of the Taiwanese cervical cancer study have significant implications for clinical practice and future research. From a clinical perspective, the observed increase in cervical cancer incidence among younger cohorts, particularly in urban areas, underscores the need for targeted screening and vaccination strategies. This trend suggests a potential shift in the age group at highest risk, warranting earlier and perhaps more frequent screening in younger populations. Furthermore, the study highlights the critical role of HPV vaccination programs, which could be expanded to include older cohorts that have not yet benefitted from such preventive measures. Additionally, timely cervical cancer prevention education for younger cohorts is imperative. For future research, the study opens avenues for exploring the sociocultural factors contributing to the rise in cervical cancer incidence among younger generations, particularly in urban settings. Investigating the impact of sexual behaviour, urbanization and healthcare access on HPV infection rates could provide valuable insights. Additionally, research could focus on the effectiveness of current screening methods in detecting early-stage cancers in younger populations. Comparative studies in different cultural and healthcare settings would also be valuable to understand the generalizability of these findings and to develop globally applicable prevention and treatment strategies.

In conclusion, in this study, we have observed a notable surge in cervical cancer risk among younger Taiwanese generations, aligning with trends in global counterparts. Such trends, deeply interwoven with sociocultural shifts like increased sexual permissiveness and urban modernization, call for enhanced preventive strategies. The introduction of HPV vaccines is promising, but its benefit mainly accrues to those born post-2004, potentially leaving a gap for those born between 1990 and 2004. Our findings accentuate the significance of organized screening, especially in detecting and managing cervical squamous cell carcinoma. The observed peak in cervical cancer risk at ages 40–44 underscores the latency of HPV infections acquired during early sexual activity years. As Taiwan marches towards the WHO’s goal of global cervical cancer elimination, a comprehensive understanding of these cohort trends is pivotal to formulating tailored preventive and therapeutic strategies.

## Supplementary Material

Appendices.pdf

## Data Availability

Data are available from the National Taiwan Cancer Registry Database published by the Health Promotion Administration, Ministry of Health and Welfare of Taiwan. Due to legal restrictions imposed by the government of Taiwan in relation to the ‘Personal Information Protection Act’, data cannot be made publicly available. Requests for data can be sent as a formal proposal to the Health Promotion Administration, Ministry of Health and Welfare of Taiwan.
