# Supplementary material for: Emerging trends in cervical cancer incidence among younger Taiwanese generations: an urban–rural comparison
Source: Ann Med. 2025 Jan 30;57(1):2458765. doi: 10.1080/07853890.2025.2458765 (PMC11784070; doi:10.1080/07853890.2025.2458765)
Supplement: Appendices.pdf [file IANN_A_2458765_SM5058.pdf]

Appendix 1. The number of invasive cervical cancer cases diagnosed through cytological or pathological examination for each year.

Year	Cytological or pathological examination (%)	Total
2000	2731 (93.7%)	2916
2001	2414 (94.6%)	2551
2002	2115 (96.4%)	2193
2003	1997 (95.4%)	2094
2004	2257 (95.2%)	2370
2005	1953 (96.4%)	2026
2006	1795 (95.1%)	1887
2007	1776 (97.3%)	1825
2008	1742 (97.5%)	1786
2009	1756 (96.3%)	1824
2010	1673 (98.0%)	1708
2011	1653 (97.2%)	1700
2012	1560 (98.2%)	1589
2013	1566 (98.2%)	1595
2014	1445 (98.1%)	1473
2015	1475 (98.4%)	1499
2016	1437 (98.8%)	1454
2017	1430 (98.7%)	1449
2018	1417 (97.9%)	1447
2019	1374 (98.8%)	1390

Appendix 2. The number of invasive cervical cancer cases under 25 years old.

Year	Invasive cervical cancer cases under 25 years old
2000	7
2001	7
2002	5
2003	5
2004	4
2005	4
2006	2
2007	3
2008	5
2009	0
2010	3
2011	3
2012	5
2013	2
2014	6
2015	7
2016	2
2017	1
2018	1
2019	5

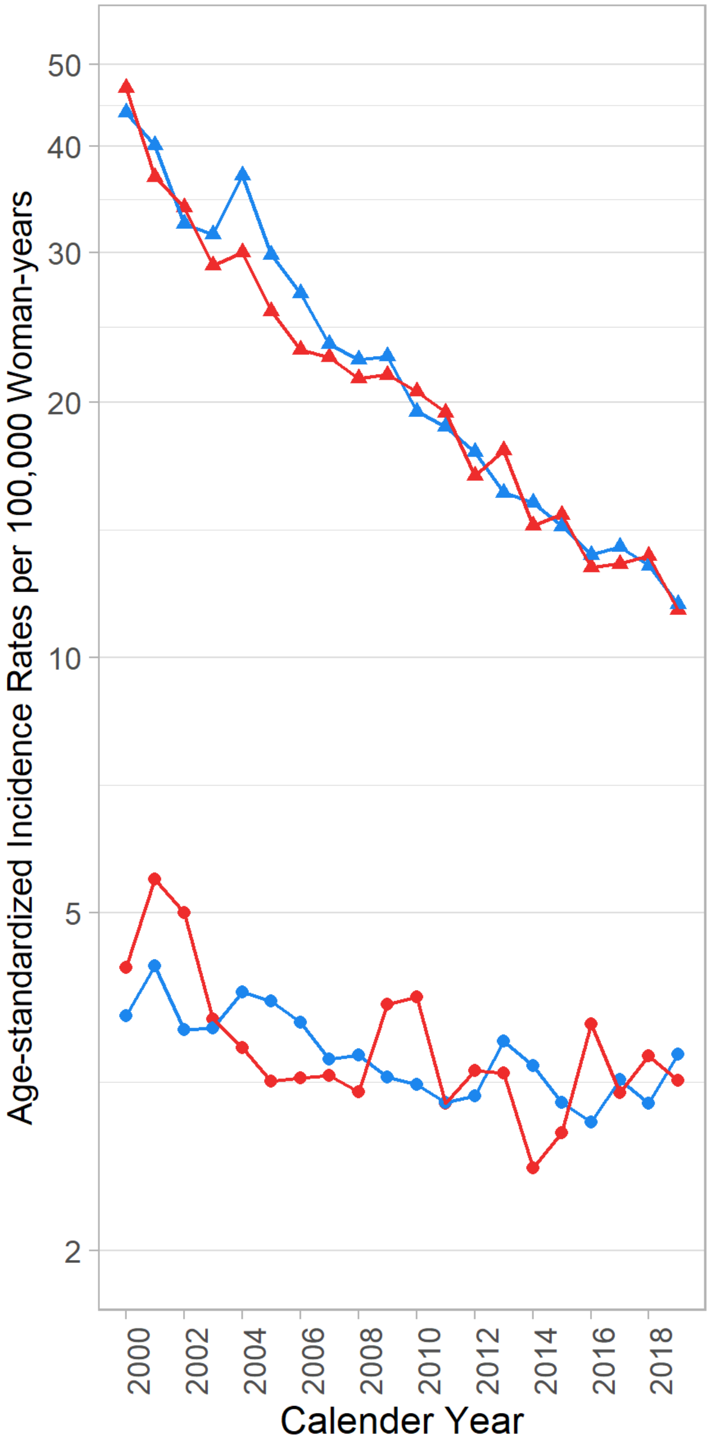

Appendix 3. Age-standardized incidence rate of adenocarcinoma and squamous cell carcinoma of cervix from 2000 to 2019 in women in Taiwan (red: urban region; blue: rural region; ●: adenocarcinoma; ▲: squamous cell carcinoma).

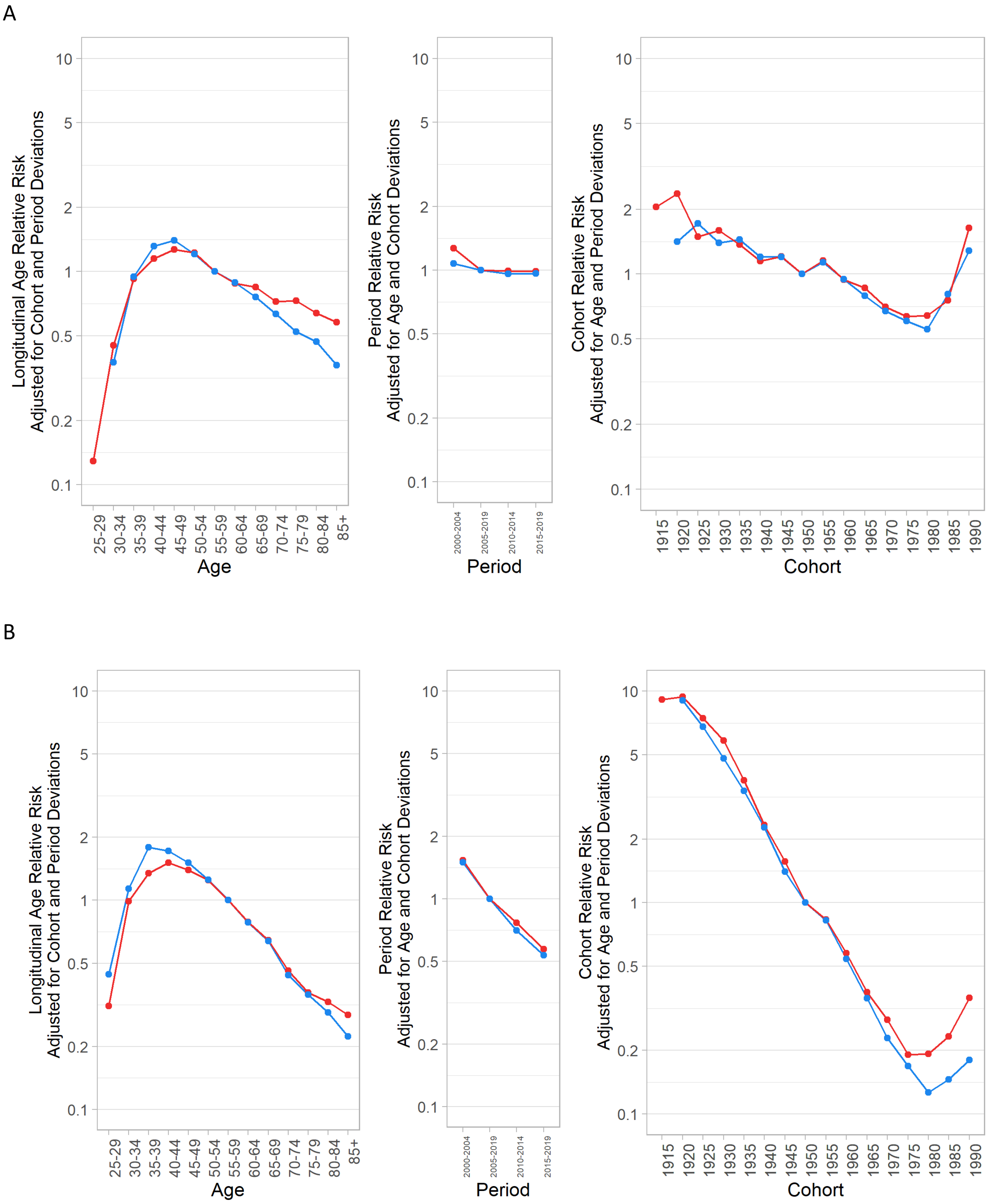

Appendix 4. Effects of age, period, and cohort on adenocarcinoma and squamous cell carcinoma of cervix incidence rates in women in Taiwan (red: urban region; blue: rural region; A: adenocarcinoma; B: squamous cell carcinoma).

Appendix 5. The number of invasive cervical cancer cases by International Federation of Gynecology and Obstetrics (FIGO) stage for each year.

FIGO stage	2004	2005	2006	2007	2008	2009	2010	2011	2012	2013	2014	2015	2016	2017	2018	2019
0	2,217	2,227	2,337	2,705	2,664	2,826	2,856	2,825	2,859	2,784	2,649	2,660	2,591	2,698	2,671	2,925
1	1	13	7	10	6	4	5	11	6	3	4	8	4	5	4	6
1A	28	22	12	19	14	18	16	22	22	14	18	22	11	11	11	15
1A1	143	131	107	157	158	152	156	153	142	138	122	104	103	94	72	73
1A2	41	20	28	20	29	35	13	35	24	22	25	11	27	19	15	15
1B	182	144	96	68	55	52	42	32	35	29	32	34	22	20	25	18
1B1	440	337	394	448	413	424	455	382	343	369	297	320	347	317	298	135
1B2	130	93	138	135	158	123	104	118	127	120	95	81	103	100	106	107
1B3	0	0	0	0	0	0	0	0	0	0	0	0	0	0	0	46
2	2	12	9	3	2	5	4	3	3	4	3	5	3	1	3	0
2A	164	143	136	150	131	142	62	45	39	24	31	30	16	14	19	10
2A1	0	0	0	0	0	0	31	35	47	34	34	42	30	38	27	23
2A2	0	0	0	0	0	0	38	43	39	40	41	51	35	40	50	27
2B	266	225	264	245	271	274	251	269	220	253	240	224	247	238	229	138
3	6	10	6	0	1	5	3	2	3	1	3	1	1	5	1	1
3A	27	27	24	27	30	20	18	16	23	28	17	23	17	23	24	14
3B	125	113	99	140	118	139	145	145	162	103	134	150	137	151	113	49
3C1	0	0	0	0	0	0	0	0	0	0	0	0	0	0	0	247
3C2	0	0	0	0	0	0	0	0	0	0	0	0	0	0	0	69
4	0	0	2	0	0	0	1	0	0	0	8	2	3	5	5	3
4A	28	32	43	48	50	58	53	60	42	54	35	38	39	45	59	42
4B	53	40	38	45	67	79	99	116	101	113	119	126	123	115	114	130
Missing	878	721	457	25	20	23	30	5	8	8	81	69	55	52	102	88
